# An assessment of mangrove forest in northwestern Mexico using the Google Earth Engine cloud computing platform

**DOI:** 10.1371/journal.pone.0315181

**Published:** 2024-12-05

**Authors:** Luis Valderrama-Landeros, Carlos Troche-Souza, José A. Alcántara-Maya, Samuel Velázquez-Salazar, Berenice Vázquez-Balderas, Edgar Villeda-Chávez, María I. Cruz-López, Rainer Ressl, Francisco Flores-Verdugo, Francisco Flores-de-Santiago

**Affiliations:** 1 Coordinación de Geomática, Comisión Nacional Para el Conocimiento y Uso de la Biodiversidad, Tlalpan, Ciudad de México, Mexico; 2 Instituto de Ciencias del Mar y Limnología, Unidad Académica Mazatlán, Universidad Nacional Autónoma de México, Mazatlán, Sinaloa, Mexico; 3 Instituto de Ciencias del Mar y Limnología, Unidad Académica Procesos Oceánicos y Costeros, Universidad Nacional Autónoma de México, Coyoacán, Ciudad de México, Mexico; Brunel University London, UNITED KINGDOM OF GREAT BRITAIN AND NORTHERN IRELAND

## Abstract

Mangrove forests are commonly mapped using spaceborne remote sensing data due to the challenges of field endeavors in such harsh environments. However, these methods usually require a substantial level of manual processing for each image. Hence, conservation practitioners prioritize using cloud computing platforms to obtain accurate canopy classifications of large extensions of mangrove forests. The objective of this study was to analyze the spatial distribution and rate of change (area gain and loss) of the red mangrove (*Rhizophora mangle*) and other dominant mangrove species, mainly *Avicennia germinans* and *Laguncularia racemosa*, between 2015 and 2020 throughout the northwestern coast of Mexico. Bimonthly data of the Combined Mangrove Recognition Index (CMRI) from all available Sentinel-2 data were processed with the Google Earth Engine cloud computing platform. The results indicated an extension of 42865 ha of red mangrove and 139602 ha of other dominant mangrove species in the Gulf of California and the Pacific northwestern coast of Mexico for 2020. The mangrove extension experienced a notable decline of 1817 ha from 2015 to 2020, largely attributed to the expansion of aquaculture ponds and the destructive effects of hurricanes. Considering the two mangrove classes, the overall classification accuracies were 90% and 92% for the 2015 and 2020 maps, respectively. The advantages of the method compared to supervised classifications and traditional vegetation indices are discussed, as are the disadvantages concerning the spatial resolution and the minimum detection area. The work is a national effort to assist in decision-making to prioritize resource allocations for blue carbon, rehabilitation, and climate change mitigation programs.

## Introduction

Mangrove forests (halophyte trees) play an important role in the ecology of tropical intertidal coastal regions as they thrive in the transition zone between land and ocean [1]. These forests are of utmost importance for understanding wetland ecology and serve as an indicator of the impact of climate change on coastal regions [2]. Although mangrove forests are often deemed undesirable for tourism and industrial development, their relevance for conservation and protection has drastically increased globally in the past two decades [3]. They are now recognized as the primary barrier against hurricane impacts [4, 5] and the main source of commercially important fisheries in the coastal zone [6].

Although mangrove forests play a crucial role in ecological and economic contexts, a considerable portion of these forests has been identified as degraded or fragmented in various regions across the globe (e.g., [7–9]. Hence, several countries have implemented long-term monitoring programs that aim to generate reliable information on the state of mangrove forests, which can then be used by various population sectors, such as those in charge of the decision-making process [10]. Typically, these programs entail systematic and continuous monitoring of variations in mangrove species distribution [11]. Consequently, it could present a considerable challenge due to the need for accurate information processing, including time-consuming data collection, storage limitations, and computation time [12].

In Mexico, the effectiveness of mangrove monitoring programs largely depends on the financial resources allocated towards them, ranging from dispersed field surveys [13] to comprehensive national studies [10]. By far, the most efficient, practical, and economical method of monitoring mangrove forests is through the utilization of data from spaceborne platforms [14, 15]. Furthermore, the availability of historical archives of previous missions has allowed it to obtain spatial data dating back to the 1970s [16, 17]. However, the processing of spatial information depends entirely on the user, and no international standard is currently in place because mangrove forests present different species and physiognomic conditions depending on the study site.

Typically, mangrove forest monitoring programs utilize vegetation indices to elucidate the phenological trends of the canopy [18]. Nowadays, a myriad of mangrove indices are available, let alone vegetation indices in general, depending on the available multispectral or hyperspectral bands from the data source, and additional indices are expected to be developed as technological advances provide increasingly accurate data at finer spatial scales [19]. For instance, the Combined Mangrove Recognition Index (CMRI) employs the Normalized Difference Vegetation Index (NDVI) and the Normalized Difference Water Index (NDWI) to detect mangrove forests [20]. This index was originally developed for the Landsat 8 sensor, and the same authors compared the CMRI against four traditional vegetation indices, obtaining a relatively decent overall accuracy of 73.43%, mainly due to the low spatial resolution of the sensor and the large number of mangrove species found in the Indian Ocean. The Modular Mangrove Recognition Index (MMRI) is a derivative of the Normalized Difference Drought Index, which employs a correlation between NDVI and the Modified Normalized Difference Water Index. The Enhanced Mangrove Vegetation Index (EMVI) uses the green band and two short-wave infrared (SWIR) bands [21], and it was designed specifically for hyperspectral data in China. However, the authors point out that its effectiveness may be limited when applied to Sentinel-2 data due to the wide wavelength range of the sensor. The Optical and SAR images Combined Mangrove Index (OSCMI) incorporate Sentinel-1 Synthetic Aperture Radar (SAR) and Sentinel-2 multispectral data. The OSCMI showed a breakthrough in sensor synergy in four regions situated off the coast of China because SAR data is unhampered by cloud cover [22]. The Mangrove Vegetation Index (MVI) is based on three bands of Sentinel-2 data, and it has been tested on the Philippine coast and obtained favorable results in separating mangroves from other vegetation types [23]. The Mangrove Forest Index (MFI) was developed using Sentinel-2 bands with a resolution of 20 meters per pixel to identify submerged mangroves along the Chinese coast [24]. Although other mangrove indices are available in the literature, we do not intend to describe each in detail. Instead, we recommend that readers consult the above references for further details.

The Comisión Nacional para el Conocimiento y Uso de la Biodiversidad (CONABIO) initiated a project in 2007 that led to the development of the Mexican Mangrove Monitoring System (MMMS) [10]. The primary goal of this endeavor is to gather information regarding the environmental status, extension, and threats to mangrove ecosystems on a national level. The data gathered by the MMMS are employed to define actions that encourage mangrove conservation and have also served as a source of valuable academic information for other studies, such as diversity [25], hydrodynamic modeling [26], soil desalinization [27], nutrients removal in water [28], spectroscopy assessments [29], and even socioeconomic endeavors [30]. Previous studies have leveraged satellite data to conduct nationwide mapping of mangrove forests [31]. Nonetheless, the widespread reliance on this approach, which entails broad thematic classifications such as a general mangrove class, highlights the necessity for a more accurate method at the species level.

Among the mangrove species of Mexico, the red mangrove *Rhizophora mangle* is highly valuable as a nesting site for migratory birds [32], and its aerial roots protect and act as a nursery area for many species [6]. Additionally, its higher productivity compared to other mangrove species has led to the development of a comprehensive distribution map for the entire North Pacific region of Mexico by CONABIO with the main aim of promoting sustainable conservation. Hence, the objective of this study was to identify the *Rhizophora mangle* and other mangrove species areas in the Northwestern Pacific region of Mexico. To achieve this goal, we calculated rates of change in the Google Earth Engine cloud computing platform using the CMRI. This information will be critical in helping to distinguish the *Rhizophora mangle* species from other mangrove classes, such as *Avicennia germinans* and *Laguncularia racemosa*, and ultimately to identify and protect this valuable coastal ecosystem.

## Materials and method

### Study area

**Fig 1:**
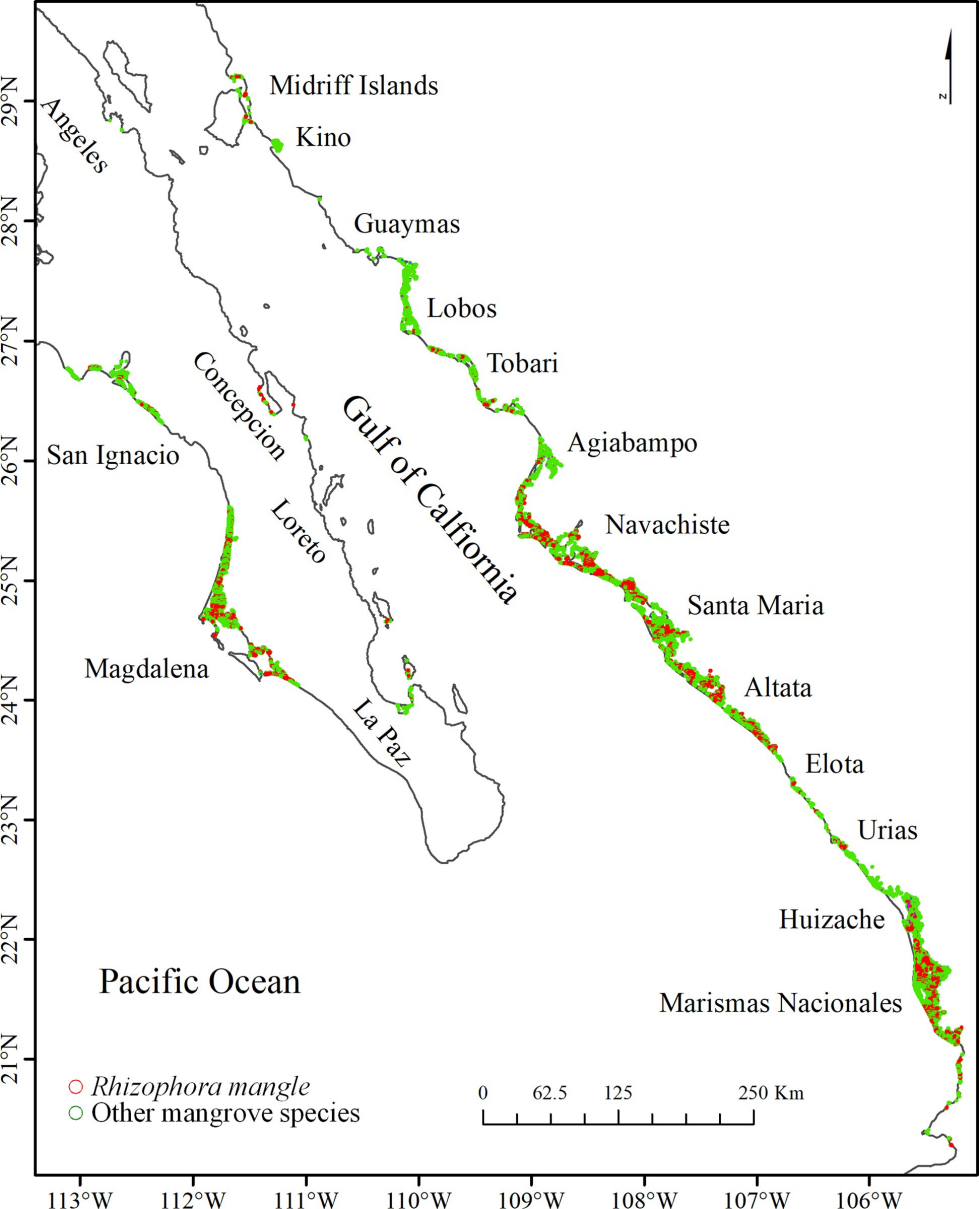
Latitudinal distribution of the two classes of mangrove forest on the northwest coast of Mexico in 2020. The mangrove classes are overlaid on the coastline from the Natural Earth website–(http://www.naturalearthdata.com/about/terms-of-use/).

The northwestern Pacific region of Mexico comprises the states of Baja California, Baja California Sur, Sonora, Sinaloa, and Nayarit (Fig 1). The coastal zone is characterized by two main conditions: the Western Pacific coast, where cold surface waters from the California Current prevail, and the Gulf of California, where the waters are considerably warmer [33]. This dichotomy in water temperature has important ecological implications as it affects the distribution of marine species [34], the productivity of fisheries [35], and possibly the extension of mangrove forests. The Gulf of California is a marginal elongated sea (265900 km^2^) that spans approximately 1000 km in length and has a variable width of around 90 to 200 km [36]. The unique geomorphology and wind-driven processes of the region generate strong vertical currents that lead to upwellings with high concentrations of dissolved nutrients toward the surface, thereby causing high primary productivity rates along the gulf [37]. Even during strong climatic events such as the El Niño Southern Oscillation, the primary productivity rates remain consistently high within the Gulf of California [38]. Despite being in a semiarid region, the eastern coast of the Gulf of California presents numerous coastal lagoons and estuaries, which are strongly influenced by the rivers originating from the western mountain range [39]. The aforementioned coast is particularly distinguished by its large extent of mangrove forests, starkly contrasting the more arid climate along the west coast [40].

In the northwestern coast of Mexico, there exist four principal species of mangroves: *Rhizophora mangle*, *Laguncularia racemosa*, *Avicennia germinans*, and *Conocarpus erectus*. These mangroves have been protected since 2010 under the official Mexican legislation NOM-059-SEMARNAT-2010. Unfortunately, the mangroves in this area are undergoing degradation primarily due to the expansion of aquaculture ponds, unauthorized logging, the development of tourist infrastructure, and modifications in hydrosedimentary patterns caused by river damming [41].

### Spaceborne data

The Sentinel-2 satellite platform from the European Space Agency was utilized for our analysis, which consists of twin satellites with 10 m spatial resolution and five-day temporal resolution, allowing for dense time series to filter out noise such as errors due to clouds and shadows [42]. The data was procured through the Google Earth Engine platform (GEE), which provides geospatial data analysis and visualization tools for academic and commercial purposes [43]. The GEE maintains an extensive database of Sentinel-2 images that facilitate pixel-by-pixel operations. However, to execute these operations, one must write a code in JavaScript or Python, with the former being the primary development language [44]. Specifically, the script is designed to collect the Sentinel-2 data based on specific dates, apply a cloud mask for quality assurance, outline the equation for generating the mangrove index, define the necessary phenological periods for characterization, produce temporal composites, and export the multi-temporal images [13]. It is important to note that the performance of these operations is subject to the platform’s conditions, as all processing is performed on the Google server [45]. Our focus was on the COPERNICUS/S2 collection, which constitutes the most extensive compilation of images from this mission since its launch in the latter half of 2015 [31]. This collection features radiometrically corrected and orthorectified radiance data, with multispectral registration at the subpixel level classified as level 2A, which offers surface reflectance data [46].

### Pre-processing, mangrove extraction, and classification

We selected a 5-year time window to reduce inconsistencies in the data caused by atmospheric conditions and establish a mangrove phenological baseline [47]. Hence, we used the available data from 2015 to 2020 to identify mangrove species groups based on seasonal changes. Regarding the presence of clouds, which is minimal in an arid/semiarid zone throughout the year, a cloud cover filter of less than 10% in GEE was used [48]. The Combine Mangrove Recognition Index [20] was calculated on pixels from all available Sentinel-2 data for the coastal zone of northwestern Mexico over five years. The CMRI equation is (NIR-Red/NIR+Red)-(NIR-SWIR/NIR+SWIR), where NIR, Red, and SWIR represent the near-infrared, red, and short-wave infrared bands, respectively. All processing was done in GEE, so no images were downloaded for this study.

We selected the CMRI from a range of mangrove indices due to the notable separation in phenological patterns observed between the mangrove classes in our study area during an initial test. Furthermore, the maximum value of the CMRI has been previously used as a representative threshold for mangrove classes in this arid region [18]. Once we separated the mangrove forests from other vegetation, the remaining maximum CMRI value was classified bimonthly using the Random Forests ensemble algorithm with 2000 random trees [49]. In this case, only a legend with two classes was utilized: (i) *Rhizophora mangle* and (ii) other dominant mangrove species (*Laguncularia racemosa* or *Avicennia germinans*). We have made this decision due to difficulties in discriminating between *Laguncularia racemosa* and *Avicennia germinans* in this part of the globe while utilizing spectroscopy [29] and multispectral data [18].

Based on the bimonthly Random Forests CMRI-based classification, which depends on the mangrove class, a series of accumulated anomalies were estimated, which consists of compiling a matrix that captures the sum of the differences, pixel-by-pixel, among each consecutive bimonthly classification and the annual average [13]. The cumulative differences are expected to increase bimonthly with each iteration in locations with changing coverage, such as those that have undergone mangrove clearance (negative process of change or loss) or colonization by seedlings (positive process of change or gain). In fact, by performing the accumulated anomaly, we ensure that the change is effective over the land cover and not a temporal anomaly due to annual phenological trends or some minimal interannual change [50]. Despite being able to generate maps of annual differences, we decided to show the overall map of change between the 2015 and 2020 anomalies. We converted the raster maps into vector format, and splinter polygons were eliminated by adjusting the minimum mappable area, which was 0.2 ha (i.e., 20 pixels in the highest resolution bands). Any patches smaller than this threshold are not included in the representation.

### Validation and accuracy assessment

The reference data for the classification was procured using in situ data from prior projects and plots. When appropriate, high-resolution images from Google Earth Pro were also employed. However, these were exceedingly uncommon and outdated, as the northwest coast of Mexico is categorized as a remote area devoid of nearby prominent urban centers. When available, aerial photographs from the Secretary of the Navy (Mexico) and Unmanned Aerial Vehicles (UAV) were also used [51]. A sample of 480 aerial photographs was gathered from helicopter flights conducted in 2015–2016 and 2020, comprising equal proportions of *Rhizophora mangle* sites and the other dominant mangrove species. The decision was to focus solely on analyzing the mangrove classes in the accuracy assessment to facilitate the spatial processing of the extensive dataset. Furthermore, excluding other thematic classes along the coastline may minimize errors in the analysis, given that not all the potential classes could be found along the entire coastal area [31]. The photographs were meticulously examined to ascertain their congruity with the recorded geographic positions. A minimum area of approximately 400 m^2^ (4 pixels of 10 m) on the photograph is deemed necessary to identify a feature in the image [52]. A confusion matrix was created to evaluate the two mangrove classes in the 480 locations. The confusion matrix provides a systematic method for evaluating the performance of a classification algorithm by facilitating a comparison between the actual classes of the testing data and those anticipated by the classification algorithm (e.g., Random Forest). The matrix consists of four essential counts: the true positives, the true negatives, the false positives, and the false negatives. Following the traditional approach, we used the above comparisons to estimate overall accuracy, user and producer accuracies, and errors of omission and commission [53]. Finally, the Kappa coefficient was also calculated to estimate the level of agreement between the classes on the map and reality while considering those that could occur by chance in the model [54].

## Results

The distribution of the two mangrove classes for 2020 exhibits a discernible latitudinal pattern (Fig 1 and S1 Fig). In particular, along the western side of the Baja California peninsula, the highest concentration of mangroves is located in Bahía Magdalena, situated between 24 and 25 degrees north, as well as in Bahía San Ignacio, positioned between 26 and 27 degrees north, which represents the northern limit of the mangrove distribution. Within the Gulf of California, we identify mangroves as far north as 29 degrees on both coasts (i.e., Baja California and Sonora); however, they are only present in a limited number of bays along the Baja California Sur coast, while they are more extensively distributed along the Sonora, Sinaloa, and Nayarit shore, specifically within numerous coastal lagoons and estuaries.

The surface area covered by *Rhizophora mangle* varies by state, with Nayarit accounting for over half of the total area (22474 ha). Sinaloa follows with 13997 ha, while Baja California Sur has a total area of 5514 ha, Sonora has 878.8 ha, and Baja California has only 1.5 ha (Table 1). There appears to be an inverse relationship between mangrove forest extent and latitude, with greater mangrove area at lower latitudes. A similar trend occurred with the other mangrove class, with Sinaloa having a higher extension of 63227.2 ha compared to the southern state of Nayarit (44130 ha). Regarding the negative process of change, Sinaloa has encountered the most extensive loss of mangrove cover (1208 ha), followed by Nayarit (575.7 ha), Sonora (27.8 ha), and Baja California Sur (5.9 ha). On the contrary, Sinaloa had the major increase in mangrove area (135.3 ha), followed by Nayarit with 29.8 ha, Baja California Sur with 18.3 ha, and Sonora with 5.4 ha. Alternatively, no loss or gain of canopy cover was reported in the small, fragmented mangrove patches of the northernmost regions of Baja California (Table in S1 Table).

**Table 1:** The surface area in hectares by mangrove class for 2020. The process of change represents both mangrove classes between 2015 and 2020.

	Class			
State	*Rhizophora mangle*	Other dominant mangrove species	Gain	Loss
Baja California	1.5	9.1	0	0
Baja California Sur	5514	20670.9	18.3	5.9
Sonora	878.8	11564.8	5.4	27.8
Sinaloa	13997	63227.2	135.3	1208.4
Nayarit	22474.4	44130.3	29.8	575.7
Total	42865.6	139602.2	208.8	1817.8

**Fig 2:**
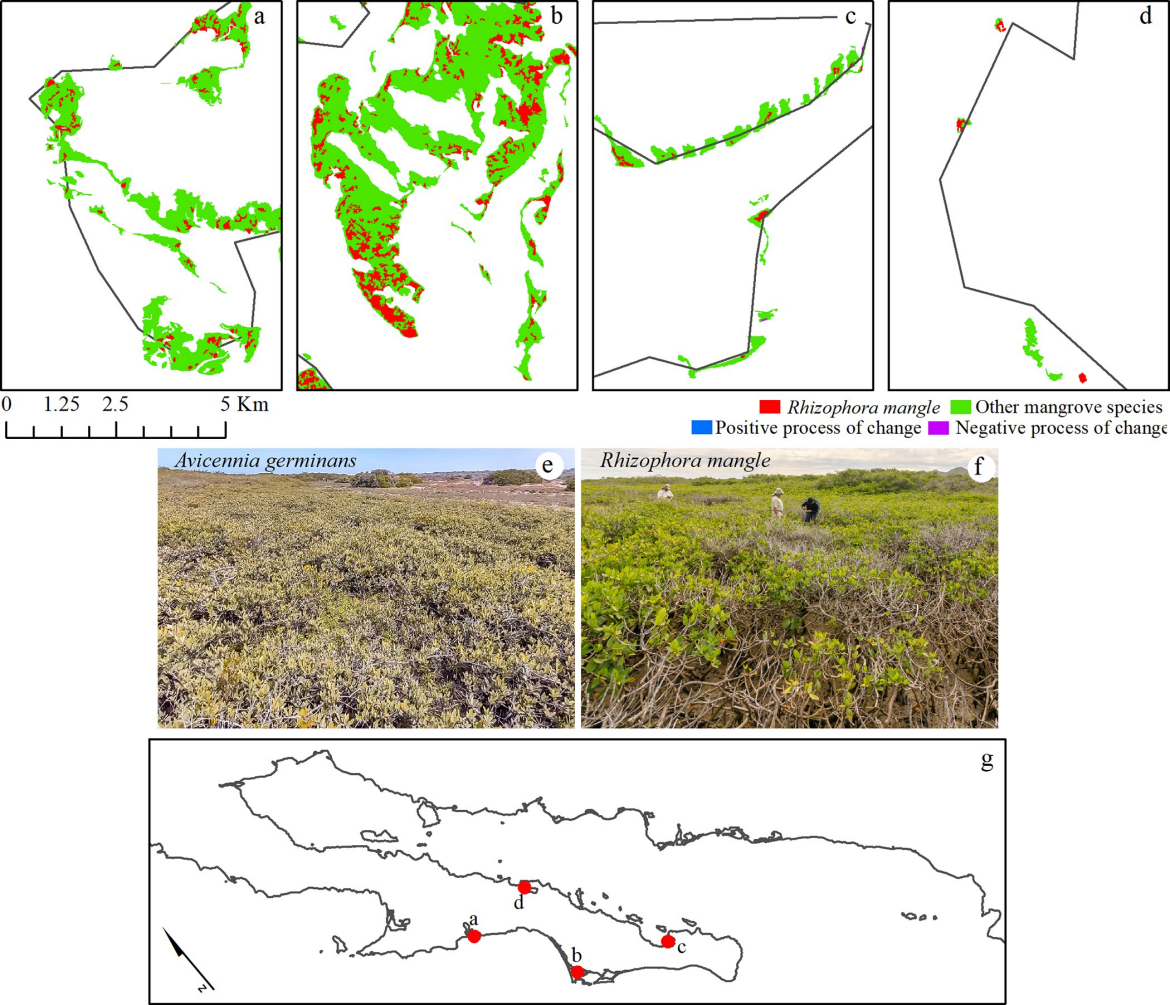
Representative portraits of four lagoon systems and bays in the Baja California peninsula. The two photographs show the typical shrubby mangrove condition of *Avicennia germinans* and *Rhizophora mangle*. The red circles on the map show the specific location of the four representative zones. The mangrove classes are overlaid on the coastline from the Natural Earth website–(http://www.naturalearthdata.com/about/terms-of-use/).

The overall accuracy of the most recent mangrove classification in 2020 is estimated to be 92%, and the Kappa coefficient resulted in 0.84 (Table 2). According to the evaluation by classes, the *Rhizophora mangle* sites have an estimated error of commission of 10% and an error of omission of 6%. In cases where other mangrove species are identified on the map, these error values are inverted, implying that the map overestimates the areas with *Rhizophora mangle* as the dominant species by 10% and omits other areas by 6%. The distribution of mangroves in the entire Baja California peninsula is typically characterized by *Rhizophora mangle* near the tidal channels and extensive patches of *Avicennia germinans* in shrub condition, less than one meter in height (Fig 2 and S2 Fig). Although there are dispersed patches of mangroves in this region, particularly in the northern area, the majority of the mangroves are situated within bays.

**Table 2:** The error matrix for the two mangrove classes in 2020.

	Reference data		
Class	*Rhizophora mangle*	Other dominant mangrove species	Total
*Rhizophora mangle*	216	14	230
Other dominant mangrove species	23	225	250
Users’ accuracy	90	94	
Producers’ accuracy	94	90	
Commission error	10	6	
Omission error	6	10	
Overall accuracy	91.9		
Kappa coeficient	0.84		

**Fig 3:**
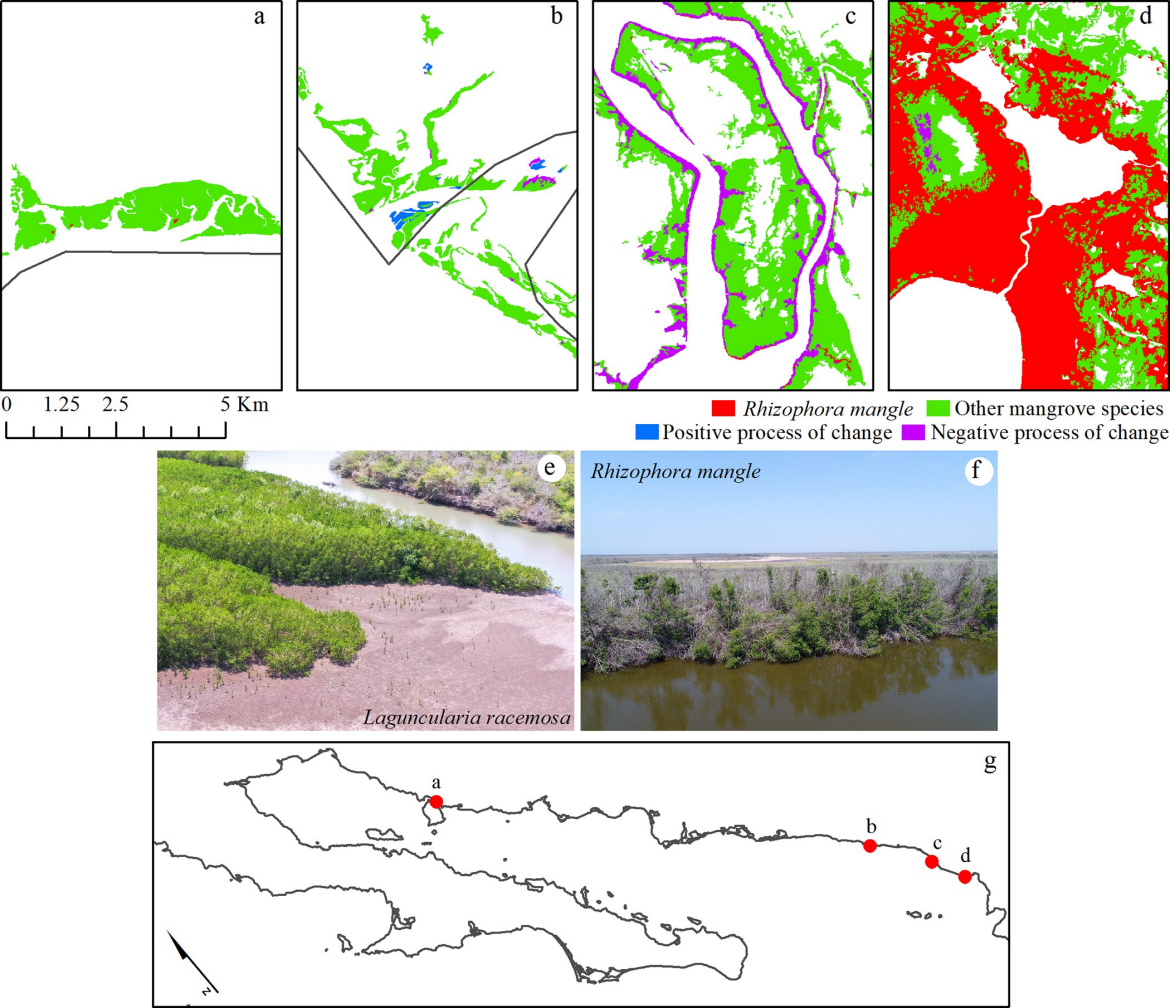
Representative portraits of four coastal lagoons and estuarine systems on the eastern coast of the Gulf of California. The two photographs show the typical mangrove conditions of the eastern coast of the Gulf of California: (e) the natural recruitment of *Laguncularia racemosa* at the mouth of the Baluarte River (positive process of change), and (f) the impact of Hurricane Willa (October 2018) on the *Rhizophora mangle* class (negative process of change). The red circles on the map show the specific location of the four representative zones. The mangrove classes are overlaid on the coastline from the Natural Earth website–(http://www.naturalearthdata.com/about/terms-of-use/).

The coastal lagoon systems associated with river mouths on the east side of the Gulf of California exhibit a major extension of mangrove forests (Fig 3 and S3 Fig). For instance, the regions located in the northernmost section show large extensions of *Avicennia germinans* in a shrub condition but much taller (3–4 m) than the same species throughout the Baja California peninsula. It is common to find monospecific *Laguncularia racemosa* forests along the riverbank of the river mouths in the southeastern region. On the other hand, the coastal lagoons in this region exhibit a combination of the three species where *Rhizophora mangle* is always located adjacent to tidal channels. Unfortunately, this coast has suffered major losses due to the impact of hurricanes and the transformation to aquaculture ponds. However, there has also been an interesting increase in mangrove extension, mainly due to the recruitment of propagules at river mouths.

## Discussion

The smallest area accurately represented on the mangrove forest distribution map produced by Sentinel-2 corresponds to 0.2 ha, which is more detailed than the minimum mappable area of 1 ha used by other previous endeavors in the same area [17]. This level of detail is necessary to identify the frequently found dispersed stands or fragmented canopy at the species level due to adverse environmental effects such as hypersaline soil conditions. However, it is worth noting that identifying *Rhizophora mangle* in the Baja California peninsula poses a major challenge, attributed to several factors, including limited accessibility to the region and the difficulties associated with obtaining field data through on-ground observations. For instance, a very recurrent problem in selecting ground truthing sites was due to security reasons. In addition, the availability of free imagery from Google Earth was scarce, and the available images resulted in very old data, which caused the number of ground truthing sites to be relatively low (i.e., 480) for such an extensive coastal region. Consequently, the area is considered highly uncertain, and there is a pressing need to secure resources for conducting additional field sampling in that part of the country. Moreover, to classify such a large area, it is imperative to have access to a high-end workstation and a dependable, high-speed network connection because a large portion of the processing will be executed in the cloud.

The scarce presence of mangroves along the west coast of the Baja California peninsula is attributed to the existence of cold-water masses from the California Current, which has a predominant north-south direction. On the contrary, the latitudinal distribution of mangroves within the Gulf of California increased by two degrees towards the north, a direct response to the water masses within the gulf, which are much warmer as it is an evaporation basin [37]. However, it is worth noting that introduced mangroves can be found in San Diego, Pacific coast of California, at 33 degrees north [55]. Hence, we anticipate that their distribution range will continue to extend further northward due to the future effects of global warming.

The results of the CMRI were not compared with other vegetation indices, let alone mangrove indices, because the final classification generated a satisfactory overall accuracy, and most of the area presents only one type of terrestrial vegetation due to its inherent arid climate. Specifically, unlike tropical regions where terrestrial vegetation or freshwater wetlands are located near the mangrove forests, in this arid region, the CMRI showed a clear signal in coastal lagoons and estuaries where mangroves thrive. This situation was verified in areas where the UAV could be deployed or higher spatial resolution images such as WorldView-2 or Google Earth Pro were available. The discrepancies in the *Rhizophora mangle* detection were minimal, with only a 10% overestimation and 6% omission observed in the Random Forests classification. In comparison, a study utilizing a single Sentinel-2 image and a decision tree classification technique in an eastern Pacific coastal lagoon resulted in an 11% overestimation for the same mangrove species [18]. As a result, the commission error obtained in our study closely aligns with the anticipated error. On the other hand, the capability of the CMRI to effectively differentiate between terrestrial vegetation and mangroves may be hindered when analyzing other mangrove species exhibiting different spectral characteristics within the canopy or in coastal regions where mangroves are in optimal physiognomic conditions [13]. In such scenarios, it may be prudent to consider evaluating the potential use of alternative mangrove indices.

The largest mangrove extension along the Pacific coast was found in Magdalena Bay because this protected area limits the impact of storm swell. However, the geomorphic conditions for most of the southern section of the peninsula are unsuitable for mangrove development due to the prevalence of coastal cliffs and coarse sandy beaches. On the other hand, the mangrove forests on the inner peninsula coast are notably sparse and fragmented due to the absence of extensive coastal lagoons. Moreover, the dry climate of the Baja California peninsula is characterized by a scarcity of fresh water, which is considered the main limitation of mangrove distribution in arid environments. Hence, preserving these isolated mangrove clusters is of the utmost importance because they are the sole source of vegetation on this arid coast. The canopy and roots of these mangroves have been known to provide essential protection for a myriad of ecological nesting birds [56] and play a key role in supporting economically important fish [34]. This isolated coast contrasts the eastern coastal regions of Sinaloa, Nayarit, and southern Sonora, where abundant rivers flow from the nearby mountain range of up to 3000 m above sea level.

The presence of *Laguncularia racemosa* in the Baja California peninsula is limited due to its preference for oligohaline conditions and minimal tidal variability [57], typically found near ephemeral river mouths of the east coast. In contrast, *Avicennia germinans* tolerates soil hypersaline conditions within arid areas [58]. Therefore, large extensions of the latter mangrove species are located throughout the study site. Meanwhile, *Rhizophora mangle* is mainly found at the edge of these areas due to its ability to withstand sudden changes in flood levels of up to 1.5 m [59]. These characteristics make each mangrove species uniquely adapted to their respective environments in this particular study area. However, it has been noted that the most substantial reduction in mangrove forest cover has occurred in Sinaloa, primarily attributable to the conversion of forested areas into aquaculture ponds [28], agricultural land [60], tourism developments [16], and the impact of Hurricane Willa in 2018 [5]. For instance, the aquaculture industry has resulted in substantial deforestation of the mangrove forest due to the construction of aquaculture ponds in areas adjacent to tidal channels where the mangrove forest thrives [61]. In this sense, shrimp farms release substantial quantities of organic matter into coastal lagoons, leading to eutrophication and the potential demise of fish and other aquatic organisms [62]. During hurricanes, high winds can lead to the defoliation of the mangrove forest canopy, with gusts reaching up to 250 km/h capable of collapsing trees [63]. These dead trunks can obstruct flood channels and disrupt the hydrological connectivity of basin-type mangrove species, ultimately increasing pore water salinity [64].

Classifying mangroves is challenging due to low spatial resolution in fragmented areas. While the PlanetScope mission affords a better resolution of 4 m/pixel compared to the 10 m/pixel of Sentinel-2, it does not make a relevant difference in identifying small mangrove patches. However, free visible WorldView-2 images (RGB only) from the Google Earth Pro platform could improve identification between Sentinel-2 and PlanetScope. In this sense, if there is a dearth of freely available WorldView-2 RGB images for a specific area, purchasing certain WorldView-2 imagery in isolated regions is probably worth it, where the logistical deployment of UAV data collection is not feasible. Nevertheless, due to technological advancements, we anticipate that free data at ~2 m/pixel will be available. For instance, a mere decade ago, accessing free images at 10 m/pixel was impossible.

In essence, for mangrove monitoring purposes at the national level, UAVs may not be practical due to their limited capacity to map only small areas (< 400 ha) despite providing centimeter-level spatial resolution data [51]. Moreover, maintaining a reasonable distance for UAV flights is necessary to avoid signal loss between the UAV and the pilot on the ground [65]. However, using novel algorithms, UAVs can provide up to 96% accuracy over mangrove canopy [66]. It must also be considered that classifying orthomosaics from UAV is complicated and demanding because each orthomosaic weighs several gigabytes, and traditional classification techniques typically used for satellite data are unfeasible. A potential alternative could be the use of SkySat images operated by Planet. The SkySat satellite constellation, consisting of 15 satellites, offers data at an ultra-high spatial resolution of 0.5 m/pixel, similar to some UAV platforms. Although these data are not freely available, existing institutional agreements could facilitate the access of these images for educational or research purposes. However, it is important to note that the present availability of SkySat scenes for isolated regions, such as the northwestern coast of Mexico, is still in its infancy.

## Conclusions

The Random Forests CMRI-based classification was formulated to illustrate the mangrove forests in the North Pacific region spanning 2015 through 2020. The methodology and data utilized are easily accessible and can be employed anywhere. Once familiar with the cloud platform, it demonstrates to be quite user-friendl. The data was utilized to produce a map that accurately identifies areas where the *Rhizophora mangle* species thrives dominantly over other species, with 92% accuracy. The map reveals that an estimated 42866 ha of *Rhizophora mangle* were present in the North Pacific region during the period under examination. One of the most notable features of the map is its depiction of the area affected by Hurricane Willa in October 2018, which caused major alterations in the structure of mangroves over 1269 ha between the states of Sinaloa and Nayarit.

The utilization of dense time series of remote sensing data has facilitated the portrayal of seasonal growth patterns in mangroves in the North Pacific area. The examination disclosed that while latitudinal gradients were apparent throughout the region, notable local variations were also observed, signifying the adaptive responses of *Rhizophora mangle* to the unique environmental conditions of the coastal region. The large local differences found throughout the study area may be due to differences in the configuration of the hydrological basins that feed the coastal lagoons and estuaries—freshwater availability. Still, specific impacts caused by anthropogenic activities cannot be ruled out. Large studies and the continuation of ecosystem monitoring projects are necessary to understand these differences, the possible processes, and the consequences behind them. Collaboration with other working groups and researchers in assessing primary productivity by measuring litterfall and soil carbon could lead to developing models relating to biomass production and its relationship to climate change.

## Supporting information

S1 Fig ArcMap file showing the latitudinal distribution of the two classes of mangrove forest on the northwest coast of Mexico in 2020. (ZIP)

S2 Fig ArcMap file showing the representative portraits of four lagoon systems and bays in the Baja California peninsula. (ZIP)

S3 Fig ArcMap file showing the representative portraits of four coastal lagoons and estuarine systems on the eastern coast of the Gulf of California. (ZIP)

S1 Table Individual polygons by state and mangrove class based on the bimonthly variability of the CMRI mangrove index. (XLSX)
